# Phase 1-2a multicenter dose-escalation study of ezatiostat hydrochloride liposomes for injection (Telintra^®^, TLK199), a novel glutathione analog prodrug in patients with myelodysplastic syndrome

**DOI:** 10.1186/1756-8722-2-20

**Published:** 2009-05-13

**Authors:** Azra Raza, Naomi Galili, Natalie Callander, Leonel Ochoa, Lawrence Piro, Peter Emanuel, Stephanie Williams, Howard Burris, Stefan Faderl, Zeev Estrov, Peter Curtin, Richard A Larson, James G Keck, Marsha Jones, Lisa Meng, Gail L Brown

**Affiliations:** 1 University of Massachusetts Medical Center, Worcester, MA, USA; 2 University of Texas Health Science Center, San Antonio, TX, USA; 3 The Angeles Clinic & Research Institute, Santa Monica, CA, USA; 4 University of Alabama at Birmingham Comprehensive Cancer Center, Birmingham, AL, USA; 5 Hematology Oncology Associates of Illinois, Chicago, IL, USA; 6 Sarah Cannon Cancer Center, Nashville, TN, USA; 7 MD Anderson Cancer Center, Houston, TX, USA; 8 Oregon Health & Science University, Portland, OR, USA; 9 University of Chicago, Chicago, IL, USA; 10 Telik, Inc, Palo Alto, CA, USA; 11 Professor of Medicine, New York Medical College, Director, MDS Program, St Vincent's Comprehensive Cancer Center, 325 West 15th Street, New York, NY 10011, USA

## Abstract

**Background:**

Ezatiostat hydrochloride liposomes for injection, a glutathione S-transferase P1-1 inhibitor, was evaluated in myelodysplastic syndrome (MDS). The objectives were to determine the safety, pharmacokinetics, and hematologic improvement (HI) rate. Phase 1-2a testing of ezatiostat for the treatment of MDS was conducted in a multidose-escalation, multicenter study. Phase 1 patients received ezatiostat at 5 dose levels (50, 100, 200, 400 and 600 mg/m^2^) intravenously (IV) on days 1 to 5 of a 14-day cycle until MDS progression or unacceptable toxicity. In phase 2, ezatiostat was administered on 2 dose schedules: 600 mg/m^2 ^IV on days 1 to 5 or days 1 to 3 of a 21-day treatment cycle.

**Results:**

54 patients with histologically confirmed MDS were enrolled. The most common adverse events were grade 1 or 2, respectively, chills (11%, 9%), back pain (15%, 2%), flushing (19%, 0%), nausea (15%, 0%), bone pain (6%, 6%), fatigue (0%, 13%), extremity pain (7%, 4%), dyspnea (9%, 4%), and diarrhea (7%, 4%) related to acute infusional hypersensitivity reactions. The concentration of the primary active metabolites increased proportionate to ezatiostat dosage. Trilineage responses were observed in 4 of 16 patients (25%) with trilineage cytopenia. Hematologic Improvement-Erythroid (HI-E) was observed in 9 of 38 patients (24%), HI-Neutrophil in 11 of 26 patients (42%) and HI-Platelet in 12 of 24 patients (50%). These responses were accompanied by improvement in clinical symptoms and reductions in transfusion requirements. Improvement in bone marrow maturation and cellularity was also observed.

**Conclusion:**

Phase 2 studies of ezatiostat hydrochloride liposomes for injection in MDS are supported by the tolerability and HI responses observed. An oral formulation of ezatiostat hydrochloride tablets is also in phase 2 clinical development.

**Trial Registration:**

Clinicaltrials.gov: NCT00035867

## Background

Myelodysplastic syndrome (MDS) is a heterogeneous group of clonal hematopoietic stem cell disorders characterized by dysplasia in one or more granulocytic, erythroid and megakaryocytic lineages, leading to ineffective blood cell production and a variable risk of transforming to acute myeloid leukemia (AML) [1-4]. The treatment options available to patients with MDS are largely based on the patient's age and their prognosis as determined by the International Prognostic Scoring System (IPSS) [5]. For patients in the low to intermediate-1 IPSS risk categories, the goal of treatment is to improve ineffective hematopoiesis while providing the appropriate supportive care. In the higher risk patients, the goal is to extend survival and delay transformation to AML.

**Figure 1:**
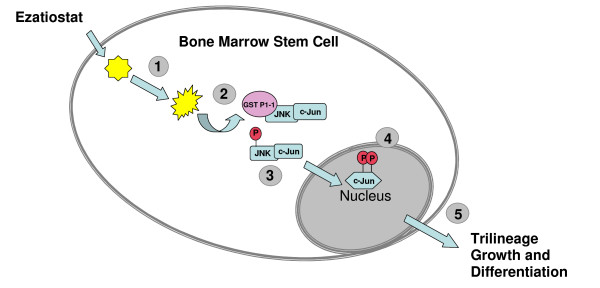
**Ezatiostat HCl Liposomes for Injection (TLK199) Mechanism of Action**. 1. Esterase action on the diester prodrug, ezatiostat liberates the active moiety, the tripeptide diacid; 2. Binding of diacid to GST P1-1 leads to release of JNK; 3. JNK phosphorylated c-JUN; 4. Phosphorylated C-Jun translocates to the nucleus and participates in transcription of growth and differentiation genes; 5. Trilineage growth and differentiation results.

Currently, there are 3 U.S. Food and Drug Administration (FDA) approved treatments for MDS; however, the need for new targeted therapies with novel mechanisms of action, such as induction of differentiation and apoptosis continue to exist. Ezatiostat hydrochloride liposomes for injection (TLK199), a novel glutathione analog, is currently being developed for the potential treatment of cytopenias associated with MDS or chemotherapy, and potentially for the treatment of MDS that has transformed to AML. Ezatiostat is a synthetic tripeptide analog of glutathione that has been shown to stimulate the proliferation of myeloid precursors [5]. Ezatiostat is metabolized to TLK117. TLK117 selectively binds to and inhibits glutathione S-transferase P1-1 (GST P1-1), an enzyme that is overexpressed in many human cancers. Glutathione S-transferase P1-1 is known to bind to and inhibit Jun-N-terminal kinase (JNK), a key regulator of cellular proliferation, differentiation and apoptosis (Figure 1) [6]. TLK117 facilitates dissociation of GST P1-1 from JNK, leading to activation of JNK and the subsequent promotion of growth and maturation of hematopoietic progenitors in preclinical models (Figure 1), while promoting apoptosis in human leukemia cell lines. Ezatiostat has been shown to stimulate the multilineage differentiation of blasts to mature monocytes, granulocytes and erythrocytes with the potential to overcome the block in differentiation (ineffective myelopoiesis) that is characteristic of MDS [5,7,8]. Mice lacking the gene for GST P1-1, due to gene deletion, when compared to wild type mice, consistently demonstrate higher than normal neutrophil levels, in addition to a significant increase in the growth rate of their embryonal derived fibroblast cells [9]. These results are consistent with the reports that GST P1-1 is a negative regulator of cellular growth and differentiation exerting its effect by binding to JNK [10]. These findings provide the rationale and scientific support for evaluation of ezatiostat in patients with MDS.

Pre-clinical data have shown that ezatiostat was well tolerated at single and repeated doses (up to 1920 mg/m^2^/day and 3200 mg/m^2^/day) in rats and dogs, respectively, with no observed dose-limiting toxicity (DLT). This first-time-in-human phase 1-2a study of the intravenous (IV) formulation was designed on the basis of safety demonstrated in multi-dose toxicology studies and efficacy reported in animal model studies. The goal of this study was to determine the maximum tolerated dose (MTD) or optimal biologic dose (OBD) [as defined as the maximum therapeutic dose which may occur at doses well below the MTD], the pharmacokinetics, safety profile and the preliminary evidence of hematologic improvement (HI) in MDS patients. The results of a phase 1-2a multicenter, multiple dose-escalation, 2 dose schedules study are reported here.

## Materials and methods

This study was conducted in accordance with International Conference on Harmonization and Good Clinical Practice standards. Institutional Review Board (IRB) approval was obtained from all participating institutions. (Note: authors Azra Raza and Naomi Galili moved to St. Vincent's Comprehensive Cancer Center, New York, NY, USA; Natalie Callander moved to University of Wisconsin Medical Center, Madison, WI; Leonel Ochoa-Bayona moved to Moffitt Cancer Center, Tampa, FL, USA, and Peter Curtin moved to University of California, San Diego, La Jolla, CA, USA; however, these institutions did not participate in this study.) All patients provided written informed consent prior to study participation.

### Patient population

Patients, age ≥ 18 years with histologically confirmed diagnosis of primary MDS with an Eastern Cooperative Oncology Group (ECOG) performance status of 0 to 2, were enrolled. Patients were required to have adequate hepatic and renal function. No prior treatment with hematopoietic growth factors within 7 days of study entry and ineligibility for allogeneic bone marrow transplantation (BMT) were other inclusion criteria. Patients with a history of allergy to eggs, leptomeningeal metastases or leukemic meningitis; chemotherapy, radiotherapy or immunotherapy within 2 weeks of study entry; use of oral corticosteroids (except for the treatment of new adrenal failure or hormones for non-MDS related conditions), and known history of hepatitis B or C, human immunodeficiency virus (HIV) infection, or an active infection requiring antibiotics were excluded.

### Study design

This phase 1-2a multicenter, open-label, multidose-escalation study of ezatiostat hydrochloride liposomes for injection (Telintra^®^, TLK199) was conducted in patients with all French-American-British (FAB) classification types of MDS. The phase 1 objectives of the study were to evaluate the safety, define the MTD or OBD, and to evaluate the pharmacokinetics of the IV liposomal formulation. In phase 1, ezatiostat was administered at a starting dose of 50 mg/m^2 ^followed by subsequent dose-escalation to levels of 100, 200, 400 and 600 mg/m^2 ^administered daily at a constant rate infusion over 60 minutes on days 1 to 5 of a 14-day treatment cycle. There are no animal models for MDS. The phase 1 dose schedule of ezatiostat administered daily × 5 every 2 weeks was based on the preclinical animal model of chemotherapy-induced neutropenia. A minimum of 3 patients were treated at each dose level. At least 2 patients must have completed 5 days of treatment and 9 days of follow-up prior to subsequent patients being enrolled at the next higher dose level. Patients who did not experience a drug-related toxicity were allowed to escalate to the next dose level after at least 1 ezatiostat-naïve patient safely completed the next higher dose level.

If no more than none of 3 or 1 of 6 patients experienced a DLT, 3 subsequent patients were enrolled at the next higher dose level. Dose-escalation continued until 2 or more patients in a cohort experienced a treatment-related DLT. A hematologic DLT was defined as a grade 4 hematologic toxicity complicated by infection, severe hemorrhage or marrow aplasia persisting greater than 4 weeks. A non-hematologic DLT was defined as any treatment-related grade 3 or 4 non-hematologic toxicity occurring during the first treatment cycle.

The MTD was defined as the highest dose at which none of 3 or 1 of 6 patients experienced a DLT or 1 full dose level below the level where a DLT was observed. If biologic activity based on the HI rate was observed prior to the MTD being established, the OBD would be selected for phase 2a evaluation. Patients were allowed to continue treatment until the patient experienced a lack of MDS response [defined as lack of hematologic improvement response after receiving 2 cycles of therapy] or unacceptable toxicities.

The objectives for the phase 2a study were to evaluate safety, determine the optimal dose schedule, and determine the objective hematologic improvement response rate by MDS International Working Group (IWG) (2000) response criteria. Patients were enrolled sequentially to the 2 dose schedules that were evaluated: ezatiostat administered IV at 600 mg/m^2 ^daily on days 1 to 5 or days 1 to 3 of a 21-day treatment cycle. In phase 2a, the 2 dose schedules were selected to test whether clinical benefit could be obtained in MDS patients on a more convenient IV dose schedule(s) to ensure the regimen could be given as an outpatient. Patients were allowed to continue treatment until MDS progression or unacceptable toxicities. In both phases of the study, adverse events (AEs) were graded in accordance with the National Cancer Institute-Common Toxicity Criteria, version 2.0 (NCI-CTC, v2.0) [11].

### Drug formulation and administration

Ezatiostat hydrochloride liposomes for injection is formulated as a sterile, white lyophilized powder and was reconstituted with 0.9% Sodium Chloride for Injection, USP (United States Pharmacopeia) and diluted in 5% Dextrose Injection, USP prior to IV administration. Each vial contains 103 mg of active substance (ezatiostat hydrochloride liposomes for injection); the reconstituted product contains 10 μg/ml of ezatiostat.

The filtered infusion solution was administered at a constant infusion rate over 60 minutes. Prior to receiving the first infusion, patients were premedicated with dexamethasone, antihistamines and an H_2 _blocker. If no acute allergic reaction occurred after the first infusion, patients received subsequent infusions without premedication, at the investigator's discretion.

### Baseline and follow-up assessments

All patients underwent a screening evaluation including a complete medical history, physical examination with vital signs, assessment of ECOG performance status, electrocardiogram (ECG) and chest X-ray. Pretreatment laboratory evaluation included complete blood count (CBC) with differential, reticulocyte count, coagulation profile, serum chemistry profile, urinalysis and pregnancy test (for female patients of child-bearing potential only).

Within 72 hours of day 1 of each subsequent treatment cycle, laboratory tests (CBC with differential and chemistry profile), a physical examination including vital signs, an assessment of ECOG performance status, documentation of concomitant medication(s) used, and an assessment of AEs were performed and documented. On days 1, 2 and 5 of the first treatment cycle, blood samples for pharmacokinetic assay of ezatiostat blood levels were obtained at specified time intervals. Concentrations of ezatiostat and the active metabolites, TLK236 and TLK117, were determined in whole blood by an LC-MS assay. On day 1 in the first treatment cycle, urine samples were also collected at pre-dose and within 24 hours following the infusion.

Complete blood count with differential were repeated daily on days 1 to 5 and day 8 of the first treatment cycle and on days 1, 5 and 8 of subsequent cycles. Hematologic improvement response assessment by IWG (2000) criteria was performed during every other treatment cycle and at the end of study treatment. A formal validated quality of life instrument was not utilized in this phase 1-2a study; however, an informal questionnaire was administered at baseline and on day 1 of each treatment cycle to assess key MDS clinical symptoms.

In phase 2, on day 1 in the first treatment cycle, patients underwent a physical examination including vital signs and an assessment of ECOG performance status, laboratory assessments (CBC with differential, reticulocyte count, chemistry profile and urinalysis), documentation of concomitant medication(s) used and assessment of AEs. Vital signs and assessment of AEs were performed on days 1 to 5 (dose schedule 1) or days 1 to 3 (dose schedule 2) of each subsequent treatment cycle. Hematologic improvement response assessment by IWG (2000) was performed every other treatment cycle and at the end of study treatment.

### Dose modifications

Patients who experienced any non-hematologic adverse event of grade 3 or higher had treatment delayed by up to a maximum of 3 weeks until recovery to grade 1 or baseline and continued treatment at a dose reduced by 20%. Patients who experienced uncomplicated drug-related grade 4 neutropenia, febrile neutropenia (except uncomplicated febrile neutropenia unassociated with grade 3 or 4 infections) and grade 4 thrombocytopenia had treatment reduced by 20% for all subsequent treatments.

For any patient who did not meet the minimum retreatment criteria on day 15 of a treatment cycle in phase 1 or on day 22 in phase 2a, administration of the subsequent treatment cycle was delayed and the toxicity was re-evaluated. If recovery did not occur after a delay of 21 days, treatment was discontinued and the patient was followed until resolution of the AE.

### Efficacy evaluation

Hematologic improvement response assessment was performed every other treatment cycle and was based on the standardized criteria for assessing MDS response as proposed by the IWG (2000) for MDS [12,13]. In addition, in phase 2a, bone marrow assessments were reviewed at 4 months for the natural history assessment per IWG (2000).

Patients with HI in the erythroid (E), neutrophil (N) and platelet (P) cell lines were summarized by each individual cell lineage as HI-E, HI-N and HI-P, respectively, based on the number of cytopenic peripheral blood cell lineages at baseline. The primary analysis was conducted under IWG (2000) criteria.

### Ezatiostat hydrochloride liposomes for injection pharmacokinetic assessment

**Figure 2:**
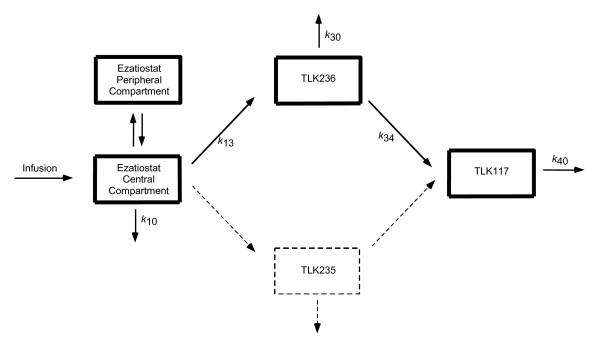
**Ezatiostat HCl Liposomes for Injection (TLK199) Pharmacokinetics**. Formation of metabolites is assumed to be unidirectional. Ezatiostat undergoes de-esterification to both TLK235 and TLK236; however, because the quantity of TLK235 measured in this study is consistently less than the level of quantification, this pathway is ignored (dashed lines). TLK236 undergoes further de-esterification to TLK117. Each of ezatiostat and TLK236 can be eliminated via more than one pathway. However, this study provides no insight into the fraction of each entity eliminated by each pathway.

Plasma and urinary concentrations of ezatiostat and its metabolites (TLK235, TLK236 and TLK117) were analyzed by an LC-MS assay. Limit of quantification (LOQ) was 10 μg/ml for all 3 entities. Figure 2 shows the proposed pharmacokinetic model of ezatiostat using non-linear mixed-effects modeling by NONMEM^®^.

### Statistical analysis

Demographic and baseline MDS disease characteristics of all treated patients were summarized descriptively. The sample size and the total number of doses administered per cycle per patient were summarized overall and for each dose level of ezatiostat administered IV at 50, 100, 200, 400 and 600 mg/m^2^.

The incidence of treatment-related AEs and clinically significant abnormal changes in laboratory results were summarized by the NCI-CTC v2.0 grades.

Pharmacokinetic data were analyzed with plasma concentration-time profiles constructed for each patient treated in phase 1. Summary statistics were generated for each individual and for each treatment group.

The HI, HI-E, HI-N, and HI-P response rates by IWG (2000) criteria, were calculated overall and by demographics and MDS disease characteristics among efficacy-evaluable patients who received at least 2 cycles of ezatiostat. Blood transfusion requirements and clinical symptom improvements were also summarized.

## Results

### Patient demographic characteristics

Fifty-four patients (35 males and 19 females) with histologically confirmed MDS were enrolled in the study and treated at 10 centers in the United States between May 13, 2002, and September 27, 2005 (Table 1). Fifty-six percent of patients had an ECOG performance status of 1 and 65% were male. Ages ranged from 22 to 90 years (median age was 70 years).

**Table 1:** Patient Demographics and MDS Disease Characteristics (N = 54)

**Age (years)**	
Median	70
Range	22–90
**Age By Decade**	**n (%)**
< 60	8 (15)
61 – < 70	19 (35)
70 – < 80	18 (33)
≥ 80	9 (17)
**Gender**	
Male	35 (65)
Female	19 (35)
**Baseline Performance Status (ECOG)**	
0	18 (33)
1	30 (56)
2	4 (7)
Unknown	2 (4)
**Race**	
Caucasian	48 (88)
Hispanic	3 (6)
Asian	1 (2)
Black	1 (2)
Other	1 (2)
**FAB Classification**	
RA	30 (56)
RARS	9 (17)
RAEB	9 (17)
RAEB-t	3 (6)
CMML	1 (2)
Unknown	2 (4)
**Baseline Karyotype**	
Abnormal	27 (50)
Normal	27 (50)
**IPSS Score**	
Low	39 (72)
Intermediate	1 (2)
High	12 (22)
Unknown	2 (4)
**Prior Therapy**	
Blood Product Support	41 (76)
r-EPO	29 (54)
G-CSF	8 (15)
Steroids	6 (11)
Thalidomide/Lenalidomide	10 (18)
Other Chemotherapy	8 (15)
5-Azacitadine	7 (13)
Vitamins	3 (6)
GM-CSF	1 (2)
**Baseline Hematologic Values**	**Median (Range)**
ANC	1.4 (0.0–27.3)
HCT	27.0 (19.3–37.8)
Hgb	9.1 (6.7–12.6)
Platelet Count	70.0 (9.0–890)

Abbreviations: RA, refractory anemia; RARS, refractory anemia with ringed sideroblasts; RAEB, refractory anemia with excess blasts; RAEB-t, refractory anemia with excess blasts in transformation; CMML, chronic myelomonocytic leukemia; IPSS, International Prognostic Scoring System; r-EPO, recombinant epoetin; G-CSF, granulocyte colony-stimulating factor; GM-CSF, granulocyte macrophage colony stimulating factor; HCT, hematocrit; Hgb, hemoglobin.

The patients treated in this study exhibited a range of FAB subtype classifications that was typical for the disease spectrum of MDS. Thirty (56%) patients had refractory anemia (RA), 9 (17%) had refractory anemia with ringed sideroblasts (RARS), 9 (17%) refractory anemia with excess blasts (RAEB); 3 (6%) with refractory anemia with excess blasts in transformation (RAEB-t); 1 (2%) chronic myelomonocytic leukemia (CMML), and 2 (4%) were unknown. There were no patients with secondary MDS on this study.

Twenty-seven (50%) patients had an abnormal karyotype. Trilineage cytopenia was present in 20 patients, bilineage cytopenia was present in 16 patients, and unilineage cytopenia was present in 18 patients.

The median number of prior therapies received by patients enrolled in this study was 1 (range 0–9) with 29 (54%) having received epoetin, 8 (15%) growth factors such as G-CSF or 1 (2%) GM-CSF, 4 (7%) lenalidomide or thalidomide, 2 (4%) azacitidine, 5 (9%) steroids, 3 (6%) other chemotherapies (e.g., amifostine, interleukin-11, premaine, investigational drug, Winrho), and 2 (4%) vitamins. At baseline, 41 (76%) patients were red blood cell (RBC) transfusion-dependent by the IWG (2000) criteria.

### Ezatiostat hydrochloride liposomes for injection study treatment administration

In phase 1, five dose levels ranging from 50 to 600 mg/m^2 ^were evaluated (Table 2). The median number of cycles received per patient was 4 (range 1–8).

**Table 2:** Ezatiostat Hydrochloride Liposomes for Injection Treatment Administration

**Phase 1**			
**Doase Cohort (mg/m^2^)**	**# of Patients**	**Total # Cycles Administered**	**Median # of Cycles per Patient (Range)**

50	6	16	3 (1–5)
100	3	10	4 (2–4)
200	3	19	7 (4–8)
400	7	36	8 (1–8)
600	7	32	5 (1–8)
TOTAL	26	113	4 (1 – 8)
**Phase 2**			

**Treatment (600 mg/m^2^)**	**# of Patients**	**Total # Cycles Administered**	**Median # of Cycles per Patient (Range)**

Dose Schedule 1	10	93	8 (4 – 17)
Dose Schedule 2	18	117	4 (1 – 19)
TOTAL	28	210	7 (1 – 19)

Dose reductions due to AEs were infrequent as only 2 patients required a dose reduction (1 each at the 50 mg/m^2 ^and 600 mg/m^2 ^dose levels). A total of 13 patients had dose delays (2 occurring at the 50 mg/m^2 ^dose level, 3 at 100 mg/m^2^, 1 at 200 mg/m^2^, 4 at 400 mg/m^2 ^and 3 at the 600 mg/m^2 ^dose level). The dose of ezatiostat was increased from 100 mg/m^2 ^to 200 mg/m^2 ^in the third treatment cycle in 1 patient, and increased from 200 mg/m^2 ^to 400 mg/m^2 ^in the fourth treatment cycle in another patient.

In phase 2a, 10 patients were treated on dose schedule 1 and 18 patients on dose schedule 2. The median number of treatment cycles received per patient was 8 (range 4–17) on dose schedule 1 and 4 (range 1–19) on dose schedule 2 for a median of 7 (range 1–19) cycles per patient in phase 2a. A total of 1345 doses were administered (Table 2).

### Safety

Ezatiostat-related hematologic adverse events were uncommon with 1 patient each (2% each) for grade 4 anemia, grade 3 anemia, grade 3 leukopenia, grade 2 leukocytosis, and grade 2 thrombocytopenia (Table 3).

**Table 3:** Hematologic and Non-Hematologic Adverse Events Related to Ezatiostat in ≥ 5% of Patients

**For All Dose Groups Combined Maximum Toxicity Grade (N = 54)**					
**Adverse Event** **(Preferred Term)**	**Grade 1** **n (%)**	**Grade 2** **n (%)**	**Grade 3** **n (%)**	**Grade 4** **n (%)**	**All Grades** **n (%)**

**Hematologic**					
Anemia	0 (0)	0 (0)	1 (2)	1 (2)	2 (4)
Leukocytosis	0 (0)	1 (2)	0 (0)	0 (0)	1 (2)
Leukopenia	0 (0)	0 (0)	1 (2)	0 (0)	1 (2)
Thrombocytopenia	0 (0)	1 (2)	0 (0)	0 (0)	1 (2)
**Non-hematologic**					
Chills	6 (11)	5 (9)	0 (0)	0 (0)	11 (20)
Back Pain	8 (15)	1 (2)	0 (0)	1 (2)	10 (19)
Drug Hypersensitivity	4 (7)	1 (2)	3 (6)	2 (4)	10 (19)
Flushing	10 (19)	0 (0)	0 (0)	0 (0)	10 (19)
Nausea	8 (15)	0 (0)	1 (2)	0 (0)	9 (17)
Bone Pain	3 (6)	3 (6)	2 (4)	0 (0)	8 (15)
Dyspnea	5 (9)	2 (4)	0 (0)	0 (0)	7 (13)
Fatigue	0 (0)	7 (13)	0 (0)	0 (0)	7 (13)
Pain in Extremity	4 (7)	2 (4)	1 (2)	0 (0)	7 (13)
Diarrhea	4 (7)	2 (4)	0 (0)	0 (0)	6 (11)
Chest Pain	2 (4)	1 (2)	2 (4)	0 (0)	5 (9)
Dizziness	4 (7)	0 (0)	1 (2)	0 (0)	5 (9)
Chest Discomfort	3 (6)	1 (2)	0 (0)	0 (0)	4 (7)
Headache	3 (6)	1 (2)	0 (0)	0 (0)	4 (7)
Vomiting	4 (7)	0 (0)	0 (0)	0 (0)	4 (7)
Abdominal Pain Upper	2 (4)	1 (2)	0 (0)	0 (0)	3 (6)
Arthralgia	2 (4)	1 (2)	0 (0)	0 (0)	3 (6)
Musculoskeletal Discomfort	3 (6)	0 (0)	0 (0)	0 (0)	3 (6)
Insomnia	2 (4)	1 (2)	0 (0)	0 (0)	3 (6)

The most common ezatiostat related non-hematologic AEs of all grades experienced by ≥ 10% of the patients (n = 54) were: chills (20%), drug hypersensitivity (19%), back pain (19%), flushing (19%), nausea (17%), bone pain (15%), fatigue (13%), pain in extremity (13%), dyspnea (13%), and diarrhea (11%) (Table 3). These events were mostly grade 1 to grade 2 and were related to acute infusion hypersensitivity reactions due to the liposomal formulation, a known side effect of liposomal drugs. Hypersensitivity reactions to the liposomal formulation of ezatiostat, in some cases, were ameliorated or prevented by use of a slower infusion rate for ezatiostat and the prophylactic administration of low-dose dexamethasone, antihistamines, and an H_2 _blocker. No DLTs were observed.

In phase 1, across all dose levels, a total of 39 serious adverse events (SAEs) were reported in 14 (54%) patients; 30 SAEs were unrelated to ezatiostat and 9 were ezatiostat treatment-related (anemia [n = 1], myocardial ischemia [n = 1], drug hypersensitivity [n = 3], cellulitis [n = 1], bone pain [n = 2], and pulmonary hemorrhage [n = 1]). In phase 2a, a total of 14 SAEs were reported in 10 patients (36%) in both dose schedules combined. Three events were ezatiostat treatment-related (anaphylactic reaction [n = 1] and drug hypersensitivity [n = 2]).

In phase 1, treatment with ezatiostat was discontinued due to AEs in 6 patients: 2 (33%) patients at the 50 mg/m^2 ^dose level, 2 (27%) patients at the 400 mg/m^2 ^dose level and 2 (27%) patients at the 600 mg/m^2 ^dose level. Four (15%) of the discontinuations were considered to be related to ezatiostat; 2 patients at the 50 mg/m^2 ^dose level, 1 patient at the 400 mg/m^2 ^dose level, and 1 patient at the 600 mg/m^2 ^dose level. In phase 2a, 2 patients (20%) on dose schedule 1 and 6 patients (33%) on dose schedule 2 were discontinued due to an AE.

No treatment-related deaths were reported in this study. Of the 12 deaths reported as unrelated to study treatment in phase 1, five were related to MDS and 6 were due to other causes. In phase 2a, no deaths were reported in the cohort on dose schedule 2; however, 2 deaths were reported in the cohort on dose schedule 1. These deaths were neither treatment-related nor due to MDS. Overall treatment-emergent adverse events for the study are shown in Table 4.

**Table 4:** Overall Hematologic and Non-Hematologic Treatment-Emergent Adverse Events in ≥ 5% of Patients

**For All Dose Groups Combined Maximum Toxicity Grade (N = 54)**					
**Adverse Event** **(Preferred Term)**	**Grade 1** **n (%)**	**Grade 2** **n (%)**	**Grade 3** **n (%)**	**Grade 4** **n (%)**	**Total** **n (%)**

**Hematologic**					
Anemia	0 (0)	3 (5.6)	11 (20.4)	4 (7.4)	18 (33.3)
Thrombocytopenia	0 (0)	1 (1.9)	2 (3.7)	5 (9.3)	8 (14.8)
Neutropenia	0 (0)	1 (1.9)	1 (1.9)	5 (9.3)	7 (13.0)
Febrile Neutropenia	0 (0)	1 (1.9)	4 (7.4)	0 (0)	5 (9.3)
Leukopenia	0 (0)	0 (0)	2 (3.7)	1 (1.9)	3 (5.6)
**Non-Hematologic**					
Diarrhea	13 (24.1)	6 (11.1)	3 (5.6)	0 (0)	22 (40.7)
Nausea	15 (27.8)	3 (5.6)	1 (1.9)	0 (0)	19 (35.2)
Fatigue	2 (3.7)	14 (25.9)	1 (1.9)	0 (0)	17 (31.5)
Back Pain	11 (20.4)	3 (5.6)	0 (0)	1 (1.9)	15 (27.8)
Bone Pain	6 (11.1)	5 (9.3)	3 (5.6)	0 (0)	14 (25.9)
Headache	10 (18.5)	4 (7.4)	0 (0)	0 (0)	14 (25.9)
Pain in Extremity	9 (16.7)	4 (7.4)	1 (1.9)	0 (0)	14 (25.9)
Chills	7 (13.0)	6 (11.1)	0 (0)	0 (0)	13 (24.1)
Vomiting	11 (20.4)	2 (3.7)	0 (0)	0 (0)	13 (24.1)
Drug Hypersensitivity	5 (9.3)	1 (1.9)	4 (7.4)	2 (3.7)	12 (22.2)
Pyrexia	11 (20.4)	0 (0)	0 (0)	0 (0)	11 (20.4)
Flushing	10 (18.5)	0 (0)	0 (0)	0 (0)	10 (18.5)
Insomnia	7 (13.0)	2 (3.7)	0 (0)	0 (0)	9 (16.7)
Oedema Peripheral	7 (13.0)	2 (3.7)	0 (0)	0 (0)	9 (16.7)
Constipation	3 (5.6)	5 (9.3)	0 (0)	0 (0)	8 (14.8)
Chest Pain	3 (5.6)	2 (3.7)	2 (3.7)	1 (1.9)	8 (14.8)
Cough	5 (9.3)	2 (3.7)	1 (1.9)	0 (0)	8 (14.8)
Dizziness	7 (13.0)	0 (0)	1 (1.9)	0 (0)	8 (14.8)
Dyspnoea	5 (9.3)	2 (3.7)	0 (0)	1 (1.9)	8 (14.8)
Arthralgia	3 (5.6)	3 (5.6)	1 (1.9)	0 (0)	7 (13.0)
Asthenia	4 (7.4)	3 (5.6)	0 (0)	0 (0)	7 (13.0)
Contusion	7 (13.0)	0 (0)	0 (0)	0 (0)	7 (13.0)
Decreased Appetite	4 (7.4)	2 (3.7)	1 (1.9)	0 (0)	7 (13.0)
Dry Mouth	5 (9.3)	1 (1.9)	0 (0)	0 (0)	6 (11.1)
Epistaxis	5 (9.3)	0 (0)	1 (1.9)	0 (0)	6 (11.1)
Tachycardia	5 (9.3)	1 (1.9)	0 (0)	0 (0)	6 (11.1)
Anorexia	3 (5.6)	1 (1.9)	1 (1.9)	0 (0)	5 (9.3)
Chest Discomfort	4 (7.4)	1 (1.9)	0 (0)	0 (0)	5 (9.3)
Dyspepsia	4 (7.4)	1 (1.9)	0 (0)	0 (0)	5 (9.3)
Dyspnoea Exertional	1 (1.9)	4 (7.4)	0 (0)	0 (0)	5 (9.3)
Ecchymosis	5 (9.3)	0 (0)	0 (0)	0 (0)	5 (9.3)
Musculoskeletal Discomfort	5 (9.3)	0 (0)	0 (0)	0 (0)	5 (9.3)
Somnolence	4 (7.4)	0 (0)	1 (1.9)	0 (0)	5 (9.3)
Urinary Tract Infection	1 (1.9)	4 (7.4)	0 (0)	0 (0)	5 (9.3)
Abdominal Pain Upper	2 (3.7)	2 (3.7)	0 (0)	0 (0)	4 (7.4)
Depression	3 (5.6)	1 (1.9)	0 (0)	0 (0)	4 (7.4)
Dry Skin	4 (7.4)	0 (0)	0 (0)	0 (0)	4 (7.4)
Infusion Site Bruising	4 (7.4)	0 (0)	0 (0)	0 (0)	4 (7.4)
Infusion Site Reaction	3 (5.6)	0 (0)	1 (1.9)	0 (0)	4 (7.4)
Neck Pain	3 (5.6)	0 (0)	1 (1.9)	0 (0)	4 (7.4)
Pneumonia	1 (1.9)	1 (1.9)	2 (3.7)	0 (0)	4 (7.4)
Pruritus	4 (7.4)	0 (0)	0 (0)	0 (0)	4 (7.4)
Rash	4 (7.4)	0 (0)	0 (0)	0 (0)	4 (7.4)
Anxiety	2 (3.7)	1 (1.9)	0 (0)	0 (0)	3 (5.6)
Conjunctival Hemorrhage	3 (5.6)	0 (0)	0 (0)	0 (0)	3 (5.6)
Dehydration	1 (1.9)	0 (0)	1 (1.9)	1 (1.9)	3 (5.6)
Dysgeusia	3 (5.6)	0 (0)	0 (0)	0 (0)	3 (5.6)
Hematuria	2 (3.7)	0 (0)	1 (1.9)	0 (0)	3 (5.6)
Hyperhidrosis	2 (3.7)	0 (0)	1 (1.9)	0 (0)	3 (5.6)
Hypertension	1 (1.9)	2 (3.7)	0 (0)	0 (0)	3 (5.6)
Hypokalemia	2 (3.7)	0 (0)	1 (1.9)	0 (0)	3 (5.6)
Hypotension	1 (1.9)	1 (1.9)	1 (1.9)	0 (0)	3 (5.6)
Infusion Site Erythema	2 (3.7)	1 (1.9)	0 (0)	0 (0)	3 (5.6)
Infusion Site Pain	3 (5.6)	0 (0)	0 (0)	0 (0)	3 (5.6)
Lung Infiltration	1 (1.9)	1 (1.9)	0 (0)	1 (1.9)	3 (5.6)
Mouth Ulceration	3 (5.6)	0 (0)	0 (0)	0 (0)	3 (5.6)
Musculoskeletal Stiffness	2 (3.7)	1 (1.9)	0 (0)	0 (0)	3 (5.6)
Oedema	2 (3.7)	0 (0)	1 (1.9)	0 (0)	3 (5.6)
Pharyngolaryngeal Pain	2 (3.7)	1 (1.9)	0 (0)	0 (0)	3 (5.6)
Upper Respiratory Tract Infection	1 (1.9)	2 (3.7)	0 (0)	0 (0)	3 (5.6)
Vision Blurred	2 (3.7)	1 (1.9)	0 (0)	0 (0)	3 (5.6)

### Pharmacokinetics

The pharmacokinetic model for ezatiostat and its metabolites (Figure 2) was derived from the concentrations of ezatiostat and its metabolites in blood of patients administered intravenous ezatiostat. Ezatiostat undergoes de-esterification to both TLK235 and TLK236. TLK235 and TLK236 undergo further de-esterification to TLK117. Pharmacokinetic parameters were estimated and derived for TLK199, TLK236 and TLK117. The ezatiostat elimination half life is 0.20 hours, an AUC/dose of 0.008 hours/L and a distribution half-life of 0.03 hours. The active metabolite TLK236 has a half-life of 2.65 hours, with an AUC/dose of 0.341 hours/L; the metabolite TLK117 has a half-life of 0.24–0.60 hours with an AUC/dose of 0.0116 hours/L. The data presented fit well with the proposed pharmacokinetic model. This pharmacokinetic population model will be further tested with ongoing patient data collection and future studies which will further refine the proposed model of pharmacokinetic parameters of ezatiostat and its metabolites, TLK236 and TLK177.

### Efficacy

Twelve (28%) patients had clinically significant improvement in at least 1 or more cell lineages in efficacy evaluable patients. The longest duration of therapy was 17 cycles on dose schedule 1 and 19 cycles in dose schedule 2 (Table 2). Clinically significant improvement was observed across all MDS FAB subtypes and in all blood cell lineages, including trilineage response in 4 of 16 patients (25%) with 3-cell line cytopenia, bilineage response in 1 of 13 patients (8%) with 2-cell line cytopenia, and unilineage response in 7 of 14 patients (50%) with single-cell line cytopenia meeting the MDS objective response criteria for HI (Table 5). Nine of 38 (24%) patients with low hematocrit/hemoglobin (anemia) had HI-E, 11 of 26 (42%) patients with WBC/ANC cytopenia had HI-N, and 12 of 24 (50%) patients with platelet cytopenia had HI-P. Patients experienced decreased RBC and platelet transfusion requirements, and in some cases leading to transfusion independence.

**Table 5:** Efficacy

**Hematologic Improvement Response Rate (IWG 2000)**				
**Baseline Cell-Line Cytopenia**			**n (%)**	
Trilineage			4/16 (25)	
Bilineage			1/13 (8)	
Unilineage			7/14 (50)	
				
**Hematologic Responses by Cell-Line Cytopenia (IWG 2000)**				
	**Number of Patients**	**Major Response** **n (%)**	**Minor Response** **n (%)**	**Total Response** **n (%)**

HI-E	38	5 (13)	4 (11)	9 (24)
HI-N	26	9 (35)	2 (8)	11 (42)
HI-P	24	4 (17)	8 (33)	12 (50)
**Hematologic Improvement (HI) Response by Patient Demographics & MDS Disease Characteristics**				
		**HI-E Response** **n (%)**	**HI-N Response** **n (%)**	**HI-P Response** **n (%)**

**Number of Patients (%)**		9/38 (24)	11/26 (42)	12/24 (50)
**Gender**				
Female		2/13 (15)	3/8 (38)	3/9 (33)
Male		7/25 (28)	8/18 (44)	9/15 (60)
**Baseline FAB**				
RA		6/20 (30)	5/12 (42)	7/11 (64)
RARS		1/6 (17)	2/4 (50)	1/1 (100)
RAEB		2/8 (25)	4/7 (57)	3/7 (43)
RAEB-t		0/3 (0)	0/3 (0)	0/3 (0)
CMML		0/1 (0)	0	0/1 (0)
Unknown		0	0	1/1 (100)
**Baseline Karyotype**				
Normal		3/20 (15)	6/16 (38)	5/14 (36)
Abnormal		6/18 (33)	5/10 (50)	7/10 (70)
				
**Transfusion Requirements and Clinical Symptoms Improvement (IWG 2000)**				
**Phase 1: Comparison of Decreased Transfusion Requirements and Clinical Symptoms Improvement**				
			**All Dose Levels Combined** **n (%)**	

50% Decreased Transfusion Requirements			3/15 (20)	
Clinical Symptoms Improvement			7/21 (33)	
**Phase 2a: Comparison of Decreased Transfusion Requirements and Clinical Symptoms Improvement by Dose Schedule**				
		**Dose Schedule 1 Days 1–5 of 21-Day Treatment Cycle** **n (%)**	**Dose Schedule 2 Days 1–3 of 21-Day Treatment Cycle** **n (%)**	**Total** **n (%)**

50% Decreased Transfusion Requirements		1/5 (20)	1/11 (9)	2/16 (13)
Clinical Symptoms Improvement		9/10 (90)	3/12 (25)	12/22 (55)

Abbreviations: RA, refractory anemia; RARS, refractory anemia with ringed sideroblasts; RAEB, refractory anemia with excess blasts; RAEB-t, refractory anemia with excess blasts in transformation; CMML, chronic myelomonocytic leukemia; IWG, International Working Group.

## Discussion

This phase 1-2a study was the first clinical study of ezatiostat hydrochloride liposomes for injection in patients with all FAB classification types of MDS.

In phase 1, patients with MDS were administered ezatiostat at doses up to 600 mg/m^2 ^IV daily for 5 days. Adverse events were generally mild to moderate in grade, with relatively few serious events reported. No DLTs were observed; therefore, the MTD was not obtained. The optimal biologic dose was determined to be 600 mg/m^2 ^and was administered on 2 schedules during phase 2a: 600 mg/m^2 ^IV on days 1 to 5 or on days 1 to 3 of a 21-day treatment cycle. Both dose schedules were well tolerated and hematologic improvement responses were observed on both schedules.

Hematologic improvement, including bilineage or trilineage responses, by IWG (2000) criteria was observed across all FAB subtypes of MDS, IPSS risk and in normal and abnormal karyotypes. Hematologic improvement was observed in patients who had failed or progressed following a range of prior therapies and supportive care regimens. Reduction of transfusion requirements or transfusion independence was reported in some cases. Improvements in bone marrow maturation and cellularity were also observed.

## Conclusion

In conclusion, further clinical investigation of ezatiostat treatment in patients with MDS is supported by the tolerability and hematologic improvement responses in all 3 cell lineages seen with intravenous ezatiostat, including independence or reduction of RBC and platelet transfusion requirements. An oral formulation of ezatiostat is being evaluated in phase 2 studies in MDS.

## Competing interests

Azra Raza has received honoraria from Celgene Corporation to serve on their speakers bureau. Peter Emanuel has been paid by Novartis in consultant/advisory board capacity. Stephanie Williams has received honoraria from Celgene Corporation and Millennium Pharmaceuticals to serve on their speakers bureau. Peter Curtin has received honoraria from MGI Pharma and Celgene Corporation to serve on their speakers bureau. Naomi Galili, Natalie Callander, Leonel Ochoa, Lawrence Piro, Howard Burris III, Stefan Faderl, Zeev Estrov, and Richard A. Larson declare that they have no competing interests.

James Keck, Marsha Jones, Lisa Meng and Gail L. Brown are employed by Telik, Inc.

## Authors' contributions

AR, MJ, and GB designed the research protocol; AR, NG, NC, LO, LP, PE, SW, HB, SF, ZE, PC, RAL were involved in treating patients and collecting data; LM conducted the statistical analysis; AR, NG, MJ, JK and GB wrote the paper with contributions from the other authors. All authors read and approved the final manuscript.
